# Correlation of clinical outcome, radiobiological modeling of tumor control, normal tissue complication probability in lung cancer patients treated with SBRT using Monte Carlo calculation algorithm

**DOI:** 10.1002/acm2.13004

**Published:** 2020-08-14

**Authors:** Sumit S. Sood, Damodar Pokhrel, Rajeev Badkul, Mindi TenNapel, Christopher McClinton, Bruce Kimler, Fen Wang

**Affiliations:** ^1^ Department of Radiation Oncology University of Minnesota Minneapolis MN USA; ^2^ Department of Radiation Medicine University of Kentucky Lexington KY USA; ^3^ Department of Radiation Oncology The University of Kansas Cancer Center Kansas City KS USA; ^4^ Highlands Oncology Group Fayetteville AR USA

**Keywords:** lung cancer, stereotactic body radiotherapy (SBRT), tumor control probability (TCP), X‐ray voxel Monte Carlo (XVMC)

## Abstract

**Purpose/Background:**

We analyzed the predictive value of non‐x‐ray voxel Monte Carlo (XVMC)‐based modeling of tumor control probability (TCP) and normal tissue complication probability (NTCP) in patients treated with stereotactic body radiotherapy (SBRT) using the XVMC dose calculation algorithm.

**Materials/Methods:**

We conducted an IRB‐approved retrospective analysis in patients with lung tumors treated with XVMC‐based lung SBRT. For TCP, we utilized tumor size‐adjusted biological effective dose (s‐BED) TCP modeling validated in non‐MC dose calculated SBRT to: (1) verify modeling as a function of s‐BED in patients treated with XVMC‐based SBRT; and (2) evaluate the predictive potential of different PTV dosimetric parameters (mean dose, minimum dose, max dose, prescription dose, D95, D98, and D99) for incorporation into the TCP model. Correlation between observed local control and TCPs was assessed by Pearson's correlation coefficient. For NTCP, Lyman NTCP Model was utilized to predict grade 2 pneumonitis and rib fracture.

**Results:**

Eighty‐four patients with 109 lung tumors were treated with XVMC‐based SBRT to total doses of 40 to 60 Gy in 3 to 5 fractions. Median follow‐up was 17 months. The 2‐year local and local‐regional control rates were 91% and and 78%, respectievly. All estimated TCPs correlated significantly with 2‐year actuarial local control rates (*P *< 0.05). Significant corelations between TCPs and tumor control rate according to PTV dosimetric parameters were observed. D99 parameterization demonstrated the most robust correlation between observed and predicted tumor control. The incidences of grade 2 pneumonitis and rib fracture vs. predicted were 1% vs. 3% and 10% vs. 13%, respectively.

**Conclusion:**

Our TCP results using a XVMC‐based dose calculation algorithm are encouraging and yield validation to previously described TCP models using non‐XVMC dose methods. Furthermore, D99 as potential predictive parameter in the TCP model demonstrated better correlation with clinical outcome.

## 1 INTRODUCTION

Despite the clinically observed low toxicity rates, hypofractionated treatments like SBRT can carry a relatively higher risk of normal tissue complication. This, along with the goal of optimizing the therapeutic ratio, necessitates the use of accurate dose modeling calculation algorithms in order to correctly deliver the prescribed dose. Treatment planning for lung SBRT patients, however, is challenging due to the involvement of small field sizes and low‐density lung medium (air). These characteristics may lead to electronic disequilibrium in the regions near low‐density heterogeneity interfaces, particularly at the periphery of small lung tumors.^1^

Many studies have demonstrated significant dosimetric differences between conventional algorithms (pencil beam type and convolution–superposition) and advanced algorithms (Monte Carlo [MC] type).^2^, ^3^, ^4^, ^5^, ^6^, ^7^, ^8^, ^9^, ^10^, ^11^, ^12^, ^13^ For example, the pencil beam algorithm with heterogeneity correction (PB‐hete) has been demonstrated to overestimate dose to the planning target volume (PTV) by up to 40%.^3^, ^4^, ^5^, ^6^ As a result, when the monitor units are established from PB‐hete calculations, the actual delivered dose is proportionately lower than the prescription dose. Monte Carlo algorithms have been considered as a complex yet more accurate method for performing dose calculations. The improved dose calculation by MC algorithms is due to its ability to accurately simulate radiation transport of secondary scatter photons and lateral electron equilibrium. MC‐based algorithms are therefore considered the gold standard and are now routinely implemented in clinical practice.

In addition to accurate dose calculation algorithms, treatment planning optimization techniques may aid radiation oncologists in enhancing the therapeutic benefit obtained by treatment with SBRT. Current clinical treatment planning optimization utilizes dose volume techniques. However, biological optimization incorporating tumor control and normal tissue complication models have demonstrated potential for reducing radiation‐induced toxicity.^14^, ^15^, ^16^ Tumor control probability (TCP) and normal tissue complication probability (NTCP) modeling in lung SBRT has demonstrated not only a dependence upon biologically effective dose (BED) and tumor size but also a reliance on the dose calculation algorithm utilized.^17^, ^18^ Reported TCP and NTCP modeling, however, has employed calculated dose based on non‐MC dose calculation algorithms which are less routinely implemented in clinical practice.^17^

In this study, we sought to analyze the correlation between clinical outcome and previously described non‐MC‐based modeling of local TCP and NTCP in patients treated with SBRT using the x‐ray voxel MC (XVMC) dose calculation algorithm. Compared to other MC methods, XVMC algorithm in Brainlab iPlan was configured for more accurate dose calculation in heterogeneous tissues through the kernels were allowed to change with the local electron density variations and significantly reducing calculation time by using the multigrid superposition method.^6^, ^7^, ^8^ Very useful for patient dose calculation in clinically realistic time. In addition, we evaluated the predictive potential of various PTV dosimetric parameters [mean dose, minimum dose, max dose, prescription dose, and dose to 95% (D95) /98% (D98)/ 99% (D99) of the target volume] after incorporation into the predictive TCP model. Observed local control rates were subsequently compared to those predicted by previously described 2 years TCP modeling as a function of biological effective dose (BED) and tumor size.^17^

## 2 MATERIALS AND METHODS

### 2.1 Study population and treatment

We conducted an IRB‐approved retrospective analysis of clinical outcomes and treatment planning data from patients treated with XVMC‐based lung SBRT at our institution from 2013 to 2016. The XVMC was based on the XVMC algorithm implemented in the Brainlab iPlan RT treatment planning system (Version, 4.1.2, Brainlab AG, Feldkirchen, Germany). All treatment plans were calculated on the MeanIP (average intensity projections of 4D‐CT) images using XVMC algorithm for heterogeneity corrections with 2.0 × 2.0 × 2.0 mm^3^ dose grid sizes, 2% variance (relative standard deviation of the mean), dose to medium and accuracy optimized for MLC modeling, whereas the MLC is modeled with full tongue‐and‐groove design. Our study population consisted of patients who underwent image‐guided lung SBRT for either T1‐T3 lung cancer or metastatic lung tumors from different primary subsites. Treatment schedules included total prescription doses of 40 to 60 Gy delivered in 3 to 5 fractions. Both peripheral and centrally treated lung tumors were included.

Follow‐up examination was conducted with regular interval CT scans to assess response to treatment. Eighteen FDG PET scans were performed, when clinically warranted, for patients with follow‐up CT scans which were indicative of possible disease progression. Local recurrence was defined as disease progression in the treated lung parenchyma based upon imaging and/or histologic confirmation.

### 2.2 Tumor control probability modeling

For TCP modeling, we utilized the size‐adjusted biological effective dose (s‐BED) modeling described by *Ohri* et al. which defines 2 years TCP as an exponential function of dose and assumes a linear inverse relationship between tumor size and BED in patients treated with SBRT using non‐MC‐based dose calculation.^17^ The equation is:

$$\text{TCP}=e^{\left[\text{BED}10‐c*L‐\text{TCD}50\right]/k}÷\;\left(1.0\;+e^{\left[\text{BED}10‐c*L‐\text{TCD}50\right]/k}\right)$$

where BED10 is the BED calculated using the Linear Quadratic Model with an alpha/beta of 10 Gy, TCD50 is the dose required to achieve 50% tumor control, where k = 31Gy corresponding to TCD50 = 0 Gy, c is a constant (10 Gy/cm) used to define the shape of the curve, and L is tumor diameter in centimeters. This model was chosen as it reflected data from a large multi‐institution study set in which 504 tumors treated with hypofractionated radiation therapy.

### 2.3 TCP model parameterization and evaluation

Due to relatively shorter follow‐up interval (median of 17 months), Kaplan–Meier curves were generated via GraphPad Prism 7.0 software in order to estimate 2‐year clinical tumor control rates for subsequent comparison to 2‐year control rates predicted by TCP modeling. Different PTV dose parameters were utilized to generate parameters specific to size‐adjusted BED values to be incorporated into TCP modeling for prediction and are listed in Table 1. The following PTV dose parameters were used: prescription dose, minimum dose, mean dose, max dose, D95, D98, and D99. We hypothesized that D99 TCP parameterization would provide the most precise correlation between predicted and observed outcome as it most accurately represents the actual dose delivered to PTV. Clinical outcome and estimated TCP association was evaluated by the Pearson’s correlation coefficient to determine the predictive power of each variable.

**Table 1:** PTV‐based dosimetric variables used in generating parameter specific BED values incorporated into the TCP model.

Parameter	Definition
sBED PTV Prescription dose	Size‐adjusted BED calculated by prescription dose to PTV
sBED PTV Minimum dose	Size‐adjusted BED calculated by minimum dose to PTV
sBED PTV Mean Dose	Size‐adjusted BED calculated by mean dose to PTV
sBED PTV Max Dose	Size‐adjusted BED calculated by maximum dose to PTV
sBED PTV D95	Size‐adjusted BED calculated by minimum dose to 95% of PTV
sBED PTV D98	Size‐adjusted BED calculated by minimum dose to 98% of PTV
sBED PTV D99	Size‐adjusted BED calculated by minimum dose to 99% of PTV

### 2.4 Normal tissue complication probability modeling

For normal tissue evaluation, we employed the Lyman NTCP Model described by Sonke *et al*. utilizing normal lung and rib DVHs and an α/β of 3 Gy fitted to predict grade 2 radiation pneumonitis and rib fracture.^19^ Patients evaluated for rib fracture included a subset population with peripherally treated tumors in which the PTV overlapped the ribs. Predicted toxicity rates were compared to observed clinically and statistically analyzed via the Chi‐squared test—two sided. The equation is:

$$NTCP=\frac{1.0}{1.0+{\left(\frac{TD50}{\mathrm{eud}}\right)}^{4.0*\gamma_{50}}}$$

where, $eud=\sum {\left[v_{i}EQD_{i}^{a}\right]}^{1/a}$ and $EQD_{i}=\frac{D*\left(\alpha /\beta +d\right)}{\left(\alpha /\beta +2\right)}$ with α/β = 3 Gy for normal lung (DVH for total lung minus ITV, that was *v_i_*), *TD*50 = 45 Gy, γ_50_ = 1.2; the parameter, and *a = 1.0*. *TD*50 represents the 50% probability of complication in 5 years after irradiation. For parallel organs such as lung and ribs “a = 1” parameter was based on already validated parameter from a multi‐institutional study by Sonke et al..^19^

### 2.5 Clinical outcome and predictors of recurrence

Local recurrence and local‐regional recurrence free survival rates were obtained for each patient. Predictors of local tumor recurrence were evaluated by cox regression analysis and stratified primary site (lung primary vs. lung metastasis), histology, location within the lung (central vs. peripheral), comorbidities, and target volume.

## 3 RESULTS

### 3.1 Patient, tumor, and treatment baseline characteristics

Eighty‐four patients with 109 either primary lung (n = 62) or metastatic lung (n = 47) tumors were treated with XVMC‐based SBRT. Treatment schedules included 3 to 5 fraction SBRT with total prescription doses of 40 to 60 Gy. Median prescription dose was 50Gy, and median fraction number was 5. Median target volume was 22.4 cc (range, 5.8−163.4cc). Median treatment duration was 11 days (range, 3‐24 days). Fifty‐five, 25, and 20 % of tumors were pathologically adenocarcinoma, squamous cell carcinoma, or other histology, respectively. The majority of treated lesions were peripherally located (88%). Baseline patient and tumor characteristics are outlined in Table 2. Treatment characteristics are outlined in Table 3.

**Table 2:** Baseline patient and tumor characteristics.

Characteristics	n (%)
Sex (n = 84)	
M	43 (51)
F	41 (49)
Age (y) (n = 84)	
Median (range)	72 (43 −89)
Risk Factors (n = 84)	
COPD	31 (37)
Smoking Pack years (n = 53)	31.6
ECOG Performance Status: Median (range)	1.1 (0‐3)
Disease state of treated lesion (n = 109)	
Primary	62 (57)
Metastatic/Recurrent	47 (43)
Histology (n = 109)	
Adenocarcinoma	60 (55)
SCC	28 (25)
Small‐cell carcinoma	2 (2)
Other	15 (14)
Presumptive	4 (4)
Tumor Classification (n = 109)	
T1a	37 (34)
T1b	14 (13)
≥T2	16 (14)
N2	1 (1)
M1	41 (38)
Target Volume (cc) (n = 109)	
Median (range)	22.4 (5.8 −163.4)
Treatment Site (no. of lesions treated, n = 109)	
RUL	38 (35)
RML	5 (5)
RLL	16 (15)
LUL	34 (31)
LLL	14 (12)
Mediastinum	2 (2)

**Table 3:** Treatment characteristics.

Characteristics	n (%)
No. Treated lesions	109
Dose Fractionation (Total Dose in Gy/fractions)	
50/5	65 (60)
54/3	29 (26)
60/5	10 (9)
48/4	2 (2)
40/5	3 (3)
Tumor Location	
Central	13 (12)
Peripheral	96 (88)
Treatment Time	
Median days (range)	11 (3‐24)

### 3.2 Clincal outcomes and predictors of recurrence

Median follow‐up time was 17 months (range, 6‐39 months). A total of ten local recurrence were identified, and local and regional control rates were 91% and and 78%, respectievly. The 2‐year actuarial local control rate was 87%. Median time to local progression was 15 months (range, 10‐36 months), and median time to regional progression was 17 months (range, 5‐36 months). Patient and tumor predictors of local recurrence are outlined in Table 4. Cox regression analysis revealed larger target volume (*P = *0.001) and the absence of COPD comordbidity (*P = *0.007) to be significant predictors of local recurrence. Histology, tumor location, and primary vs. metastatic tumor status did not significantly predict for local recurrence.

**Table 4:** Patient‐ and tumor‐related predictors of local recurrence.

Patient and tumor predictors of local tumor recurrence	*P*‐value (Cox Regression Analysis)
Gender (M/F)	0.310
COPD Hx (No)	0.007
Disease status of treated lesion	
Primary	0.765
Metastatic	0.257
Recurrent	0.214
Histology	
Adenocarcinoma	0.619
SCC	0.987
Other	0.876
Location (Central vs. Peripheral)	0.912
Target Volume (cc)	0.001

### 3.3 TCP and variable parameterization

**Fig. 1:**
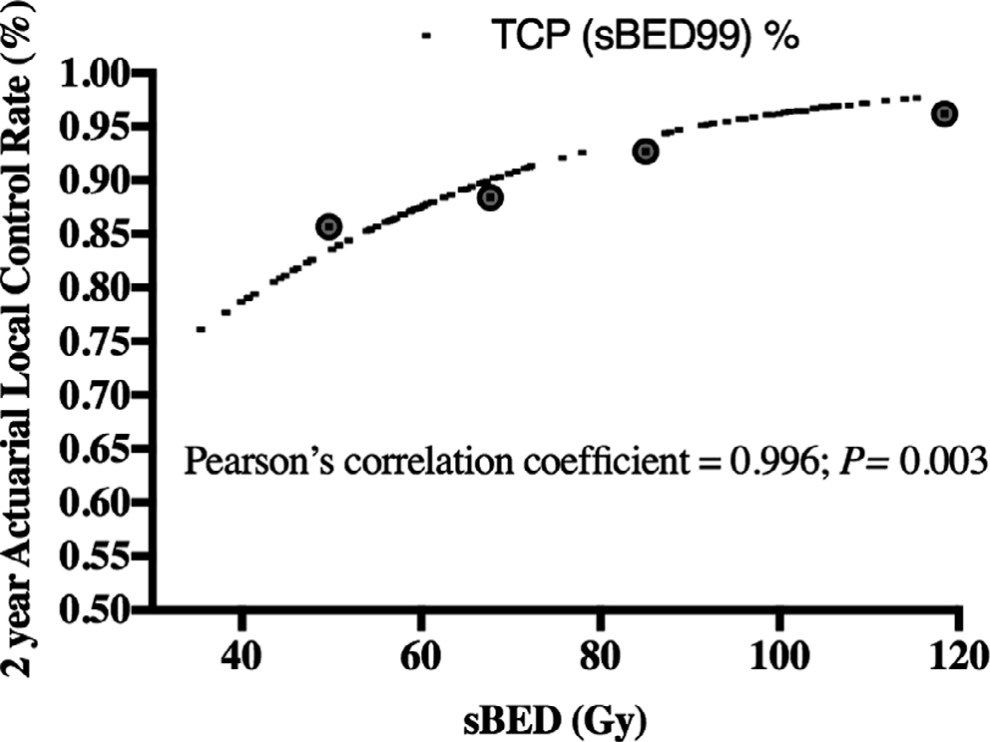
Two‐year actuarial control rates vs. size‐adjusted biologically effective dose (sBED) taking into account tumor diameter in centimeters. Each gray circle represents a mean sBED (51.0 ± 8.7; 67.0 ± 6.7; 84.1 ± 4.2; 117.2 ± 9.7) for a cohort of 26 to 28 treated tumors and their corresponding mean actuarial 2‐year local control rate. The dashed line represents the tumor control probability (TCP) as calculated by the optimal PTV dose parameter D99, representing the minimum dose delieved to 99% of the PTV. This figure demonstrates the strong correlation between observed and predicted tumor control values (Pearson’s correlation coefficient = 0.996, *P = *0.003).

Correlation of TCP modeling according to specific parameterization vs. 2‐year actuarial local control rates as evaluated by Pearson’s correlation coefficient are represented in Table 5. The following were estimated 2‐year local control rates (%) as predicted by TCP modeling based on accounting for tumor diameter and different PTV dose parameters: s‐BED Prescription dose (91% ± 2%), s‐BED Minimum PTV dose (82% ± 10%), s‐BED Mean PTV Dose (95% ± 4%), s‐BED Max PTV Dose (98% ± 2%), s‐BED D95 (91% ± 6%), s‐BED D98 (89% ± 7%), and s‐BED D99 (88% ± 7%). All TCP estimated outcomes using size‐adjusted BED (assuming linear decrease in BED with increase in tumor diameter) correlated significantly with 2‐year actuarial generated control rates (*P *< 0.05). Pearson’s correlation coefficient between TCP and observed tumor control according to PTV dosimetric parameter were the following: 0.965 for mean dose (*P = *0.035), 0.968 for minimum dose (*P = *0.031), 0.953 for max dose (*P = *0.047), 0.972 for prescription dose (*P = *0.027), 0.972 for D95 (*P = *0.028), 0.971 for D98 (*P = *0.029), and 0.996 for D99 (*P = *0.0035). The optimal parameter was determined to be D99 which exhibited a Pearson’s correlation coefficient of 0.996 and *P* value of 0.003. Plot of strong correlation between observed and predicted tumor control values calculated by the optimal PTV dose parameter D99 is demonstrated in Figure 1.

**Table 5:** Analysis correlating clinical tumor control rates to predicted control by TCP modeling based on different PTV dose parameters.

TCP Parameter	Local Control (%)	Pearson's correlation coefficient	*P*‐value
2 years Actuarial (Kaplan Meier)	87		
sBED Prescription dose	91 ± 2	0.973	0.0271
sBED Minimum PTV dose	82 ± 10	0.964	0.0351
sBED Mean PTV Dose	95 ± 4	0.964	0.0351
sBED Max PTV Dose	98 ± 2	0.953	0.0469
sBED D95	91 ± 6	0.972	0.0276
sBED D98	89 ± 7	0.971	0.0295
sBED D99	88 ± 7	0.996	0.0035

### 3.4 NTCP parameterization

Observed normal tissue complication rate did not significantly differ from those estimated by modeling (*P* > 0.05). The incidence of grade 2 pneumonitis vs. predicted was 1%(n = 1) vs. 3%, and the incidence of rib fracture (n = 21) was observed in 10% (n = 2) vs. predicted was 13%. The two patients who experienced rib fracture received dose fraction schedules of 60 Gy in 5 fractions with maxiumum point doses around 70Gy.

## 4 DISCUSSION

### 4.1 TCP and NTCP parameterization

Predicted tumor control probability correlated significantly with clinically observed local control for all size‐adjusted BED parameters (Table 5; *P < *0.05). These results support the literary work by Ohri et al. who not only demonstrated the importance of incorporation of tumor size into the model to more accurately predict observed local control rates but also determined that the probability of tumor control was significantly overestimated without taking into account tumor size.^17^ Results of multivariable analysis in our study further reinforced local control rates to be dependent on target volume size (*P = *0.001), and the importance of tumor size on treatment outcome after SBRT has been echoed clinically by several other authors.^20^, ^21^, ^22^ Although symptomatic COPD statistically correlated to local recurrent rate in this study, the similar finding has not been demonstrated in the literatures. In general patient’s COPD status is not a contraindication for SBRT. However, it could be a risk factor for development of radiation‐induced pneumonitis (RIP). In a retrospective study from Inoue et. al., they analyzed 136 Stage I lung cancer patients with COPD who underwent SBRT. There was no significant difference in overall survival or cause‐specific‐survival between patients with and without COPD. Multivariate analysis showed that COPD was statistically significant risk factors for the development of prolonged minimal RIP.^23^

In the paper by Ohri et al.,^17^ TCP was generated by using the prescription dose prescribed to PTV with subsequent generation of an associated BED value using the linear quadratic equation with an α/β value of 10. While prescription dose reflects dose delivered to the PTV, it often does not represent the most accurate approximation to actual dose delivered to the PTV. Thus, in an effort to further improve modeling prediction, we evaluated the predictive potential of several different dosimetric parameters, all of which were different dose measurements of the planning target volume. Variables utilized in generating specific BED parameters to be incorporated into the TCP model included prescription dose, minimum dose, mean dose, max dose, D95, D98, and D99. We had hypothesized that D99 TCP parameterization would have highest correlation with clinical outcome as it most accurately represents dose delivered to the PTV.

All of TCPs estimated with size‐adjusted BED parameterization did significantly correlate with clincal outcome. Among all of the size‐adjusted BED parameters, quantitatively TCP generated with D95, D98, and D99 seemed to more accurately predict than the other variables. Conversely, quantitatively minimum PTV dose and max PTV dose seemed to under and over predict TCP, respectively. We attempted to isolate the predictive potential of one parameter over the others by analyzing the Pearson’s correlation coefficient. TCP generated with D99 demonstrated the highest correlation amongst all the variables. This was in accordance with our initial hypothesis and reflects the need to inocoporate actual dose delivered to the PTV for more robust probability modeling.

Our overall incidence of normal tissue toxicity was low, including one patient with clinically observed grade 2 pneumonitis and two patients with rib fracture. Our estimated complication probablity did not significantly differ from clinically observed rates of toxicity. While this is encouraging, our low incidence of events is a limiting factor and certainly these results must be validated in larger data sets.

### 4.2 Limitations, patient population, clincal outcomes, and predictors of recurrence

There are several limitations to our analysis that must be considered. Our overall study population, local recurrent events, and follow‐up interval were limited. We experienced a total of ten local recurrences at a median follow‐up interval of 17 months. Despite having a limited follow‐up time, this time interval was likely adequate for 2 years TCP modeling and was accounted for by generating 2 years Kaplan–Meier actuarial observed local control rates. However, it does not allow for extrapolation beyond 2 years during which long‐term local recurrences may be observed.

In our sample population, we included a heterogeneous group of patients in terms of tumor size and location, patient baseline characteristics, and treatment history. As a result, the TCP model may not be generalizable to a unique group of patients. One notable difference between our study population and that included by Ohri and colleagues was the fact that we included both primary and metastatic lung tumors as compared to primary lung tumors alone. Although in our multivariable analysis evaluating predictive factors of recurrence did not identify tumor origin to be a statistically significant factor, local control after SBRT has been demonstrated to be unfavorable in metastatic lesions when compared to primary lung tumors.^24^ Our observed local and local‐regional recurrence rates at our median interval time were higher than typically expected at our median follow‐up interval. This could potentially be explained by characteristics inherent to our heterogeneous patient population. In our study cohort, 38% of patients had metastatic lung tumors. In addition, 14% of primary lung tumors had T2 disease, which have demonstrated higher rates of local recurrence compared to T1 primary lung tumors post‐SBRT in the literature.^25^

Furthermore, we only included patients treated with commonly utilized 3‐5 fraction SBRT fractionation schemes, and as a result the TCP model may not be generalizable to patients treated with single fraction or larger hypofractionated regimens.

### 4.3 Future directions

Certainly, validation of our predictive modeling experience in larger advanced dose calculation SBRT data sets is necessary to confirm our observations. Subsequently, comparison to alternative predictive models may help generate the optimal predictive model tool. In an effort to improve the therapeutic ratio in patients treated with lung SBRT, refinement of tumor control modeling may help facilitate bridging between modeling and clinical implementation with the end goal of biologically based treatment plan optimization.

In the future, we hope to further evaluate normal tissue complication probability modeling in patients who undergo MC‐based lung SBRT looking specifically at both lung parenchymal and rib toxicity.

## 5 CONCLUSION

Despite a relatively shorter follow‐up interval, our tumor control probability results using a MC‐based dose calculation algorithm are encouraging and yield validation to previously described predictive models using non‐MC dose calculation methods. Our results verify TCP modeling for lung SBRT as a function of BED and tumor size. Furthermore, we present for consideration D99 as another potential predictive parameter in the TCP model for better correlation with clinical outcome. Longer follow‐up interval and larger data sets are needed to validate our observations.

## CONFLICT OF INTEREST

The authors have no conflict of interest.
